# Exposure to second-generation antipsychotics increases circulating small extracellular vesicles that promote insulin resistance in hepatocytes

**DOI:** 10.1007/s13105-026-01250-7

**Published:** 2026-11-03

**Authors:** Vitor Ferreira, Rosa Alén, Dora Koller, Adrián Povo-Retana, Diana Grajales, Yolanda Sanchez-Carro, Lisardo Boscá, Manuel Izquierdo, Pilar López-García, Paz Garcia-Portilla, Francisco Abad-Santos, Irma Garcia-Martinez, Ángela M. Valverde

**Affiliations:** 1 Instituto de Investigaciones Biomédicas Sols-Morreale (IIBM), Consejo Superior de Investigaciones Científicas-Universidad Autónoma de Madrid., Madrid, Spain; 2 Centro de Investigación Biomédica en Red de Diabetes y Enfermedades Metabólicas Asociadas (CIBERDEM), Instituto de Salud Carlos III, Madrid, 28029 Spain; 3 Clinical Pharmacology Department, Hospital Universitario de La Princesa, Universidad Autónoma de Madrid (UAM), Instituto de Investigación Sanitaria La Princesa (IIS-Princesa), Madrid, Spain; 4 Faculty of Psychology and Health Sciences, Madrid Open University, Madrid, Spain; 5 Centro de Investigación Biomédica en Red en Enfermedades Cardiovasculares (CIBERCV), Instituto de Salud Carlos III, Madrid, Spain; 6 Centro de Investigación Biomédica en Red de Salud Mental (CIBERSAM), Instituto de Salud Carlos III, Madrid, Spain; 7 Department of Psychiatry, Faculty of Medicine, Universidad Autónoma de Madrid, Madrid, Spain; 8 Department of Psychiatry, University of Oviedo, Psyhiatric Service of Servicio de Salud del Principado de Asturias (SESPA), Instituto de Investigación Sanitaria del Principado de Asturias (ISPA), Instituto Universitario de Investigación en Neurociencia del Principado de Asturias, Oviedo, Spain; 9 Centro de Investigación Biomédica en Red de Enfermedades Hepáticas y Digestivas (CIBERehd), Instituto de Salud Carlos III, Madrid, Spain

**Keywords:** Second-generation antipsychotics, Aripiprazole, Olanzapine, Small extracellular vesicles, Insulin resistance, Inflammation

## Abstract

Second generation antipsychotics (SGAs) are widely prescribed for the management of psychiatric disorders, yet their use is associated with obesity, insulin resistance, and impaired glucose homeostasis. Small extracellular vesicles (sEV) are mediators of inter-tissue communication in metabolic diseases. Herein, we investigated the impact of the SGAs aripiprazole (ARI) and olanzapine (OLA) on circulating sEV in healthy volunteers and patients with first-episode psychosis, as well as the effects of these sEV on hepatocyte insulin signalling and systemic inflammation. Twenty-four healthy volunteers received OLA or ARI for five days. Seven individuals with first-episode psychosis were treated with SGAs; three were followed longitudinally for 6 months. sEV were isolated from plasma or culture media of SGA-treated human macrophages (h-mac) from donors. Huh7 human hepatocytes were exposed to sEV and insulin signalling was assessed. Inflammatory responses were evaluated by determining IκBα or *IL1B* expression in h-mac. In healthy volunteers, ARI significantly increased circulating sEV levels, which positively correlated with LDH, GGT, and free T_4_ plasma levels. sEV from ARI-treated subjects impaired hepatocyte insulin signalling in most cases, whereas sEV from OLA-treated patients produced milder effects. sEV from SGA-treated h-mac induced hepatocyte insulin resistance, accompanied, particularly with ARI, by reduced IκBα and increased *IL1B* expression in h-mac. Patients showed elevated baseline circulating sEV and increased baseline *IL1B* expression in PBMCs compared to healthy volunteers, and SGAs further enhanced these alterations. Our results suggest that elevations in circulating sEV associated to disease and SGA treatment may contribute to insulin-desensitization and inflammation, two hallmarks of metabolic dysfunction.

**Graphical abstract:**

**Figure:**
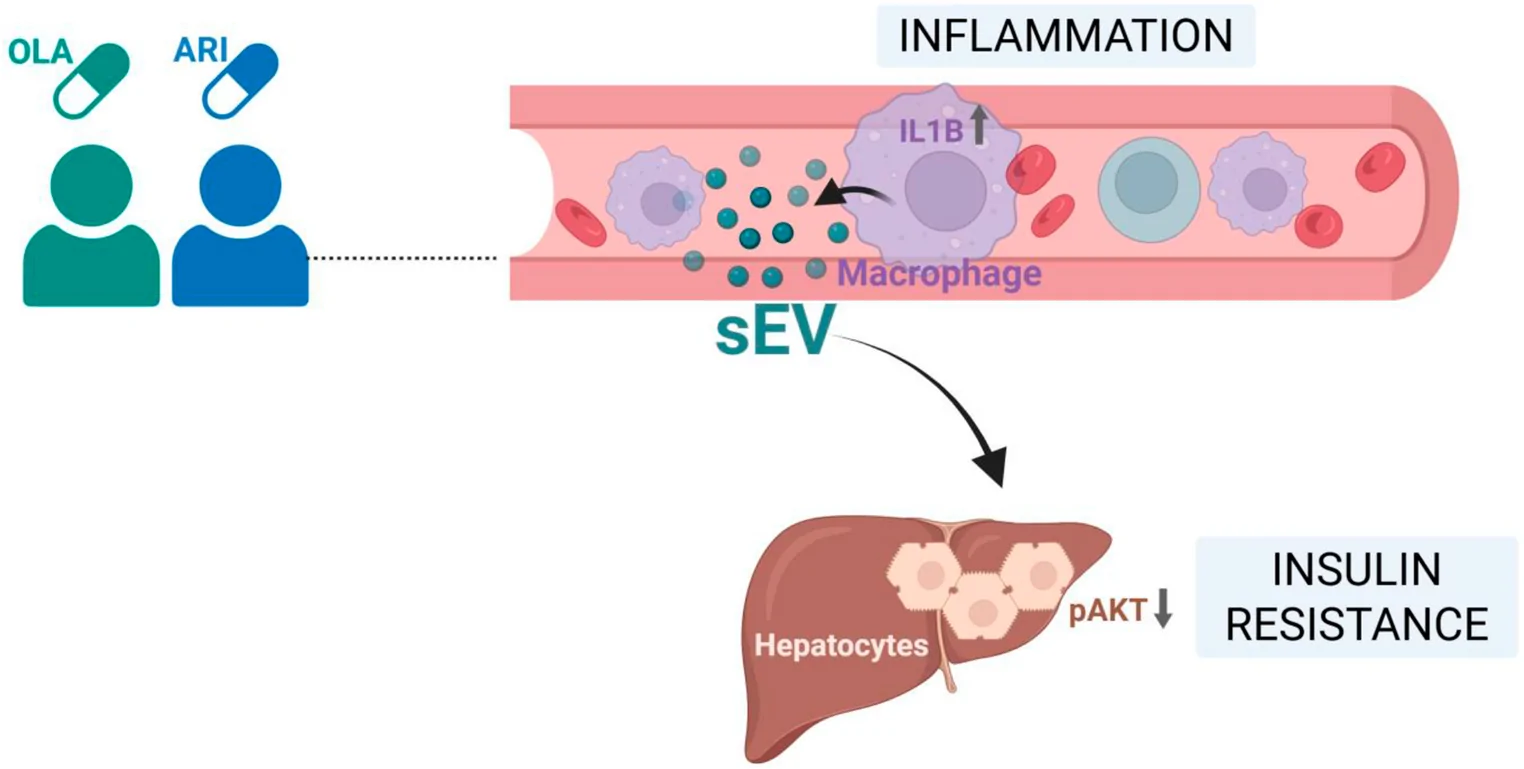

**Supplementary Information:**

The online version contains supplementary material available at https://doi.org/10.1007/s13105-026-01250-7.

## Keypoints

In healthy volunteers ARI induces a higher rise in circulating sEV than OLA

Circulating sEV from ARI-treated volunteers attenuate hepatocyte insulin signalling

sEV from ARI-treated h-mac increase *IL1B *expression and promote hepatocyte insulin resistance

First-episode psychosis elevated plasma sEV and SGAs enhanced this effect

**Supplementary Information:**

The online version contains supplementary material available at https://doi.org/10.1007/s13105-026-01250-7.

## Introduction

Schizophrenia is a severe chronic psychiatric disorder affecting approximately 21 million people worldwide. It typically manifests in late adolescence or early adulthood and is characterized by positive symptoms such as delusions, hallucinations, cognitive impairment and/or negative symptoms including apathy, avolition, alogia and anhedonia [1, 2]. This symptomatology significantly impair patients’ stability, quality of life, and social functioning. Studies indicate that severe mental illness is associated with a 10–20 year reduction in life expectancy compared to the general population, with cardiovascular disease being the leading cause of death in individuals with schizophrenia [3–5].

Although pharmacological treatment is generally effective in controlling positive symptoms, it has only a limited impact on the long-term course of schizophrenia [6]. As a result, overall recovery rates continue to be notably low, comparable to those observed when Emil Kraepelin first characterized the condition [7]. Current clinical guidelines recommend second-generation antipsychotics (SGAs), such as olanzapine (OLA) and aripiprazole (ARI), as first-line therapy [8, 9] due to their improved efficacy and lower risk of extrapyramidal side effects compared to first-generation agents. However, SGAs are frequently associated with metabolic disturbances, including weight gain, hyperglycemia, and dyslipidemia, which affect a substantial proportion of patients [10–12].

A 2018 meta-analysis review of 11 clinical trials in healthy volunteers showed that SGAs can directly induce insulin resistance and weight gain independently of disease-related factors. These early alterations may progressively lead to type 2 diabetes and cardiovascular complications [13], ultimately reducing treatment adherence and increasing healthcare costs [14, 15]. These findings underscore the need to elucidate the molecular mechanisms underlying SGA-induced metabolic dysfunction, particularly alterations in insulin signalling and glucose and lipid homeostasis. Furthermore, investigating tissue-specific responses and inter-organ communication pathways is essential to expand the understanding of the systemic metabolic side effects associated with SGA therapy.

Small extracellular vesicles (sEV) have emerged as key mediators of intercellular communication in health and disease [16, 17]. By transferring proteins, lipids, and regulatory RNAs, sEV are capable of rapidly reprogram signalling pathways in recipient cells [18]. Of relevance, the investigation of sEV as non-invasive prognostic biomarkers has gained considerable attention, particularly in the field of oncology. For instance, sEV-derived RNA sequencing analyses from serum and urine of patients with cholangiocarcinoma, a highly aggressive biliary cancer, have demonstrated promising diagnostic potential [19]. Importantly, accumulating evidence shows that sEV released from metabolically stressed tissues propagate insulin-resistance phenotypes, impairing insulin receptor substrate (IRS)-phosphatidylinositol 3-kinase (PI3K)-protein kinase B (AKT) signalling, reducing glucose uptake, and altering lipid handling [20]. Moreover, a pioneering study by Kornek and colleagues first reported a correlation between circulating leukocyte-derived sEV and the severity of metabolic dysfunction-associated steatotic liver disease (MASLD) [21]. These findings highlight the broader applicability of sEV as biomarkers across diverse pathological conditions.

Despite this growing interest, the contribution of sEV to SGA-induced metabolic dysfunction, and to the development of insulin resistance, remains poorly understood. Given the high prevalence of metabolic side-effects in patients receiving SGAs, elucidating these mechanisms is of particular importance. In this study, we aim to decipher the impact of the SGAs OLA and ARI on the circulating sEV pool in healthy volunteers and in patients with a first-episode psychosis, as well as the downstream effects of these vesicles on hepatocyte insulin signalling and systemic inflammation, two hallmarks of metabolic dysfunction.

## Methods

**Healthy Volunteers: description and sample collection.** The study population consisted of 24 healthy volunteers (12 men and 12 women) enrolled in a phase I, multiple oral dose, open-label, randomized, crossover, two-period, two-sequence, single-center, comparative clinical trial as previously reported [22]. Participants received either 10 mg/day ARI tablets or 5 mg/day film-coated OLA tablets for 5 days (Laboratorios Alter, Madrid, Spain). The hospitalization period lasted 5 days, beginning 1 h before the first dose and ending 24 h after the final administration. The drug was administered at 9 a.m. each day under fasting conditions. After a 28-day washout period, each volunteer received the alternative treatment. The random allocation sequence, the recruitment of participants and their assignment to interventions were performed by investigators at the clinical trials unit. The study protocol was approved by the Research Ethics Committee (CEIC) of Hospital de La Princesa (registration number 3411), authorized by the Spanish Drugs Agency, and conducted in accordance with the guidelines of Good Clinical Practice and the Declaration of Helsinki (clinical trial registry name, registration number, URL and date: TREATMENT-HV; EUDRA-CT 2018-000744-26; https://reec.aemps.es/reec/public/detail.html; 23 May 2018). All participants provided written informed consent before inclusion and were free to withdraw from the study at any time. Inclusion and exclusion criteria, age, body weight, height, body mass index (BMI), as well as biochemical and hematological analyses performed at days 1, 3 and 6 after the first dose of SGAs, have been previously reported [22, 23]. sEV levels were correlated with these clinical parameters.

**Sample collection from individuals with a first psychotic episode.** A randomized clinical trial was conducted in seven patients diagnosed with first psychotic episode to evaluate their early response to antipsychotic treatment and its potential impact on metabolic dysfunction. Eligible participants were men and women aged 18–65 years, newly diagnosed, and not receiving any pharmacological treatment at enrolment or during the preceding month. Patients were recruited in mental healthcare centers by psychiatrists and were randomized with either OLA or ARI. Blood samples were collected at baseline (before treatment) and subsequently at 1 week, 1 month, and 6 months after treatment initiation to isolate peripheral blood mononuclear cells (PBMCs) and plasma-derived sEV. Of the seven patients initially enrolled, only three completed the 6-month follow-up.The study protocol was approved by Comité Ético de Investigación Clínica (CEIC) of Hospital de La Princesa (Acta CEIm nº06/18) authorized by the Spanish Drugs Agency, and conducted in accordance with the guidelines of Good Clinical Practice and the Declaration of Helsinki (clinical trial registry name, registration number, URL and date: TREATMENT-PATIENTS; EUDRA- Nº EudraCT 2018-000178-31; https://rec.aemps.es/reec/public/detail.html; 23 May 2018). All participants provided written informed consent before inclusion and were free to withdraw from the study at any time.

**PBMCs isolation**, **differentiation into macrophages and in vitro treatment with SGAs.** PBMCs were obtained from buffy coats of healthy anonymous donors in accordance with the Institutional and Centro de Transfusiones de la Comunidad de Madrid agreements (28504/000011) and processed at IdiPAZ (Madrid, Spain). All donors provided written informed consent following the ethical principles of the 1975 Declaration of Helsinki and the guidelines of the Human Subjects Committee of La Paz University Hospital (HULP; PI-3521). PBMCs were isolated by layering buffy coats over Ficoll (17–0300, Sigma-Aldrich-GE) and then centrifuging for 25 min at 450xg at RT without brake. Plasma and PBMC fractions were collected from the upper-aqueous phase of the Ficoll gradient and PBMCs washed twice with sterile phosphate buffer saline (PBS) by centrifuging for 5 min at 300xg at room temperature (RT). Residual erythrocytes from the PBMC fraction were lysed by adding diluted red blood cell lysis buffer (07850, STEMCELL, Vancouver, Canada) followed by washing with sterile PBS twice. After that, PBMCs were centrifuged for 5 min at 300xg and resuspended in serum-free Dulbecco’s Modified Eagle Medium (DMEM) (41966‐029, Gibco) supplemented with 100 U mL^− 1^ penicillin and streptomycin (15140–122, Gibco). Cells were seeded at 1–2 × 10^6^ cells per well in 6‐well culture plates or 10 × 10^7^ cells in 100 mm dishes (353 046, Falcon). Cells were maintained in serum‐free medium for 1 h to allow monocyte cell adhesion. Cells were then washed twice with PBS to remove non-adherent cells, and medium was replaced with DMEM supplemented with 10% EV-depleted fetal bovine serum (FBS). Monocytes were maintained in these conditions until human macrophages (h-mac) differentiation was evident by microscopy observation, typically after 7 days in culture. Cells were then incubated with 12.5 µM OLA or 6.25 µM ARI for different time points: 30 and 60 min for IκBα assessment by Western Blot, 8 h for *IL1B* expression analysis by real-time polymerase chain reaction (RT-qPCR), and 24 h for subsequent isolation of small sEV from conditioned medium.

**Isolation of** **PBMCs from healthy volunteers treated with SGAs and from patients with first-episode psychosis**. Fresh blood from healthy volunteers was collected at T0 (baseline), T1 (5 h), and T2 (5 days) after SGA administration. Fresh blood from patients with first-episode psychosis was collected at baseline (before treatment) and 1 week, 1 month, and 6 months after treatment protocol initiation. Blood was collected in EDTA-treated tubes and subsequently processed to obtain plasma and PBMCs. PBMCs were isolated using Ficoll gradient centrifugation as described above. The resulting cell fraction was used for total RNA extraction, followed by quantification of *IL1B* expression by RT-qPCR.

**Isolation of circulating sEV from plasma.** Plasma collected from the blood samples of healthy volunteers at T0, T1 and T2, or patients with first-episode psychosis (baseline and 1 week, 1 month, and 6 months after treatment initiation), was diluted 1:1 in PBS and centrifuged at 3,000×g for 30 min at 4 °C to remove cells, platelets, and residual debris prior to sEV isolation. The supernatant was then processed by sequential differential ultracentrifugation to isolate sEV, according to the protocol reported by Thery et al. [24], with minor modifications. This approach yields a preparation corresponding to the sEV fraction, consistent with recent reports employing comparable ultracentrifugation-based isolation methodologies [25, 26]. Briefly, plasma samples were diluted with Hanks’ Balanced Salt Solution (HBSS, 14175095, Gibco, Waltham, MA, USA) and sequentially centrifuged at 10,000×g for 45 min and 110,000×g for 70 min at 4 °C using a 60Ti fixed-angle rotor (Beckmann-Coulter, Brea, CA, USA). Pellets were resuspended in PBS (10010-023, Gibco, Waltham, MA, USA) and ultracentrifuged again at 110,000×*g* for 70 min at 4 °C in a TLA-100 fixed-angle rotor (Beckmann-Coulter, Brea, CA, USA). Each final sEV pellet isolated from the same volume of plasma (3 ml) was resuspended in 200 µl of PBS and used for further characterization or functional assays.

**sEV isolation from human macrophages-conditioned medium**. sEV were isolated from h-mac-conditioned medium using the ExoQuick Ultra TC kit (EQULTRA-20TC-1, SBI) according to the manufacturer’s instructions. After 24 h of SGA treatment, conditioned medium was collected and centrifuged at 3,000×g for 15 min to remove cells and debris, followed by an optional 12,000×g centrifugation for 10 min when residual debris was present. The clarified medium was mixed with ExoQuick-TC reagent at a 4:1 ratio and incubated overnight at 4 °C. Samples were then centrifuged at 3,000×g for 10 min, and the resulting pellet was resuspended in Buffer B. For purification, 200 µl of Buffer A were added and the sample was loaded onto the ExoQuick Ultra column after removing the storage buffer by centrifugation at 1,000×g for 30 s. The column was washed twice with 500 µl of Buffer B, sealed, and equilibrated with 100 µl of Buffer B before adding the sample. After mixing for 5 min at RT, purified sEV were eluted by removing the bottom closure and centrifuging at 1,000×g for 30 s. The final sEV fraction was stored at − 80 °C until analysis.

**sEV characterization.** sEV isolated from both plasma and h-mac-conditioned medium were characterized according to the Minimal Information for Studies of Extracellular Vesicles (MISEV2018) guidelines established by the International Society for Extracellular Vesicles (ISEV) [27].

**Nanoparticle tracking analysis (NTA).** sEV concentration (particles/ml) was determined by NTA using a Nanosight LM10 (Malvern Panalytical, Malvern, UK) equipped with fast video-capture and particle-tracking software to analyse sEV isolated from equal volumes of plasma. For each sample, at least 4 videos of 60 s containing more than 200 detected tracks per video were recorded and analysed using the NTA 2.3 software with camera level 13 and detection threshold 3 settings. Results represent the average sEV concentration obtained from the analysis of the four videos. For healthy volunteers, values are expressed as fold change relative to the T0 baseline. For patients with first-episode psychosis, sEV levels are expressed as total particles × 10¹⁰/mL.

**Western Blot.** Western blot analysis was performed to confirm the identity and purity of the sEV preparations. Samples were evaluated for canonical sEV markers including CD63, CD81 (both under non-reducing conditions) and TSG101. Calnexin, an established endoplasmic reticulum marker, was included as a negative control to evaluate the purity of the vesicle preparation isolated from culture medium.

**Transmission Electron Microscopy.** Briefly, sEV preparations resuspended in PBS were fixed in 2% paraformaldehyde (PFA), applied to Formvar carbon-coated electron microscopy grids, post-fixed with 1% glutaraldehyde, contrasted with uranyl-oxalate solution, embedded in a mixture of uranyl acetate and methylcellulose, air dried, and visualized with a JEOL JEM 1010 electron microscope.

**Culture of Huh7 cells and stimulation assays.** Authenticated, mycoplasma-free human Huh7 hepatoma cells were maintained in DMEM supplemented with 10% heat-inactivated FBS, 100 U/ml penicillin, and 100 µg/ml streptomycin. For stimulation assays, cells were seeded in 12-well plates at 1.5 × 10⁶ cells per well and allowed to adhere for 24 h in low-glucose DMEM containing 10% FBS. Circulating sEV were isolated from equal plasma volumes (3 ml per donor), and the resulting pellet was resuspended in an equal volume of PBS (200 µl). A 40-µl aliquot of this suspension was added per well for stimulation. h-mac-derived sEV, obtained from the culture media of 40 × 10⁶ h-mac, were resuspended in 300 µl of PBS and 50 µl of the final preparation were used for stimulation. All stimulations were performed in DMEM supplemented with 2% EV-depleted FBS to minimize background vesicle contamination. After 24 h of sEV exposure, Huh7 cells were serum-starved for 1 h to synchronize insulin responsiveness and then stimulated with 10 nM insulin (I-0516, Sigma) for 10 min. Cells were subsequently harvested for downstream analyses of insulin signalling.

**Protein Extraction and Western Blot.** To obtain total cell lysates, adherent cells were scrapped and incubated for 10 min on ice in lysis buffer (10 mM Tris pH 7.5, 5 mM EDTA, 50 mM NaCl, 30 µM sodium pyrophosphate, 50 mM sodium fluoride (NaF, S7920, Sigma-Aldrich, San Louis, M, USA), 100 µM o-vanadate sodium (S6508, Sigma-Aldrich), 1% Triton X-100, 1 mM phenylmethylsulfonyl fluoride (PMSF, P7626, Sigma-Aldrich, San Louis, MO, USA), and 10 µg/ml protease inhibitors (P8340, Sigma-Aldrich, San Louis, MO, USA), pH 7.4–7.6. Lysates were clarified by centrifugation (15,600 x g for 20 min at 4 °C), and supernatants were stored at − 20 °C. Protein concentration was determined using Bradford reagent (500-0006, Bio-Rad, Hercules, CA, USA) for Huh7 cells or BCA assay (23227, Thermo Fisher Scientific, Waltham, MA, USA) for sEV samples. Protein samples were boiled at 95 °C for 5 min in loading buffer (100 mM Tris pH 6.8, 10% glycerol, 4% sodium dodecyl sulfate (SDS), 0.2% bromophenol blue and 2 mM β-mercaptoethanol) and submitted to 10–12% SDS-PAGE. For CD63 and CD81 detection, samples were processed under non-reducing conditions. Proteins were transferred onto methanol-activated polyvinylidene difluoride membranes (IPVH00010, Merck Millipore, Burlington, MA, USA), blocked with 4% BSA or 5% non-fat dry milk for 2 h, and incubated overnight at 4 °C with primary antibodies. After washing, membranes were incubated with HRP-conjugated secondary antibodies, and immunoreactive bands were visualized with ECL chemiluminescent substrate (170–5061, Bio-Rad, Hercules, CA, USA) on a ChemiDoc imager (733BR-3548, Bio-Rad, Hercules, CA, USA). Densitometric analysis of the bands was performed using ImageJ 2.0 Software (National Institutes of Health (NIH), Maryland, USA). The complete list of antibodies used is provided in Table S1.

**RNA extraction and RT-qPCR.** Total RNA was extracted from PBMCs or h-mac using TRIzol reagent (AM9738, Invitrogen, Madrid, Spain) and reverse-transcribed with the SuperScript First-Strand Synthesis System (18080051, Invitrogen) following the manufacturer’s instructions. RT-qPCR was performed on an ABI 7900 Sequence Detector (Applied Biosystems, Massachusetts, USA). The primer sequences used were for *IL1B*, forward 5’-TGAGCACCTTCTTTCCCTTCA and reverse 5’-ATGCTGCACATAAGCCTCGTT; and for *ACTB*, forward 5’-CTGGAACGGTGAAGGTGACA and reverse 5’-AAGGGACTTCCTGTAACAATGCA.

**Statistical analysis.** Statistical analyses were performed using GraphPad Prism 8.4.2 software (San Diego, CA, USA) and specific statistical details are provided in each figure legend. Normality of all datasets was assessed using the Shapiro-Wilk test. For normally distributed data, group comparisons were performed using one-way ANOVA followed by Bonferroni’s post hoc test, whereas non-normal datasets were analysed using the Kruskal-Wallis test for comparisons involving more than two groups or the Mann-Whitney U test for two-group comparisons. Correlations were initially explored using simple linear regression; linearity was evaluated through scatter plots and the coefficient of determination (R²), and normality of each variable was again assessed using the Shapiro-Wilk test. Correlations that did not meet linearity (R² < 0.1) or normality (Shapiro-Wilk *p* < 0.05) criteria were analysed using Spearman’s rank correlation, whereas Pearson correlation was retained when both assumptions were satisfied. Data are expressed as the mean ± SEM, and a p value < 0.05 was considered statistically significant.

## Results

**Fig. 1:**
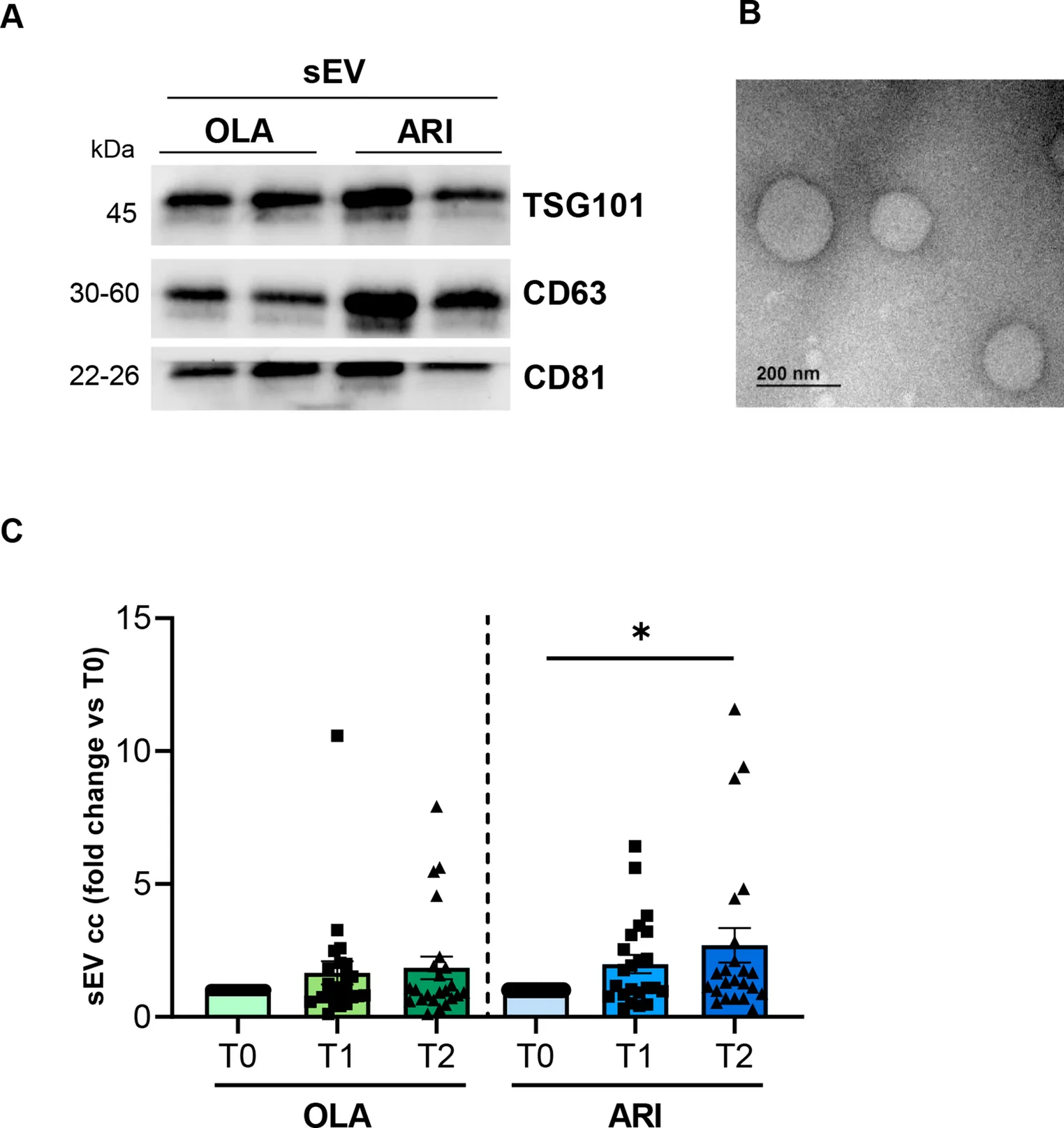
Characterization and quantification of isolated sEV from healthy volunteers after OLA and ARI treatment. **A** Representative Western blot analysis of the sEV markers TSG101, CD63, and CD81 in sEV isolated from two healthy volunteers treated with OLA or ARI. **B** Representative transmission electron microscopy image showing round vesicle-like structures with typical sEV morphology (scale bar: 200 nm). **C** Nanoparticle Tracking Analysis of sEV concentration (cc), expressed as fold change relative to T0, for OLA- and ARI-treated volunteers (*n* = 23) at time points T0 (baseline), T1 (5 h post-dosing), and T2 (5 days post-dosing). Data are presented as mean ± SEM. *p < 0.05, One-way ANOVA, Kruskal-Wallis test

**Differential effects of ARI and OLA on circulating sEV concentration in healthy volunteers.** We first evaluated the impact of OLA and ARI administration on circulating sEV levels in healthy volunteers. A total of 24 participants received either OLA or ARI, and blood samples were collected at baseline (T0), and after 5 h (T1) and 5 days (T2) of SGA exposure. sEV were isolated from platelet-free plasma and characterized in accordance with MISEV guidelines. As shown in Fig. 1A, the isolated sEV expressed canonical markers including TSG101, CD63 and CD81, and exhibited the expected round, membrane-bound morphology with sizes consistent with sEV populations, as confirmed by TEM (Fig. 1B). Quantitative analysis of sEV concentration revealed distinct temporal patterns between the two treatments (Fig. 1C). In the OLA-treated group, there was a trend towards an increase in plasma sEV concentration relative to baseline (T0) levels, although it was not statistically significant. In contrast, ARI treatment induced a significant increase in circulating sEV concentration at T2 compared to baseline. Notably, no sex-related differences were observed (Figure S1). NTA confirmed well-defined size distribution profiles characteristic of sEV populations, with no secondary peaks indicative of contamination, supporting the purity and consistency of the sEV preparations (Figure S2).

Collectively, these findings indicate that, although OLA and ARI generate comparable circulating sEV populations in terms of marker expression and morphology, only ARI treatment significantly increases their abundance in healthy volunteers. This observation suggests that the two SGAs may differentially modulate the mechanisms underlying sEV biogenesis and/or release.

**Fig. 2:**
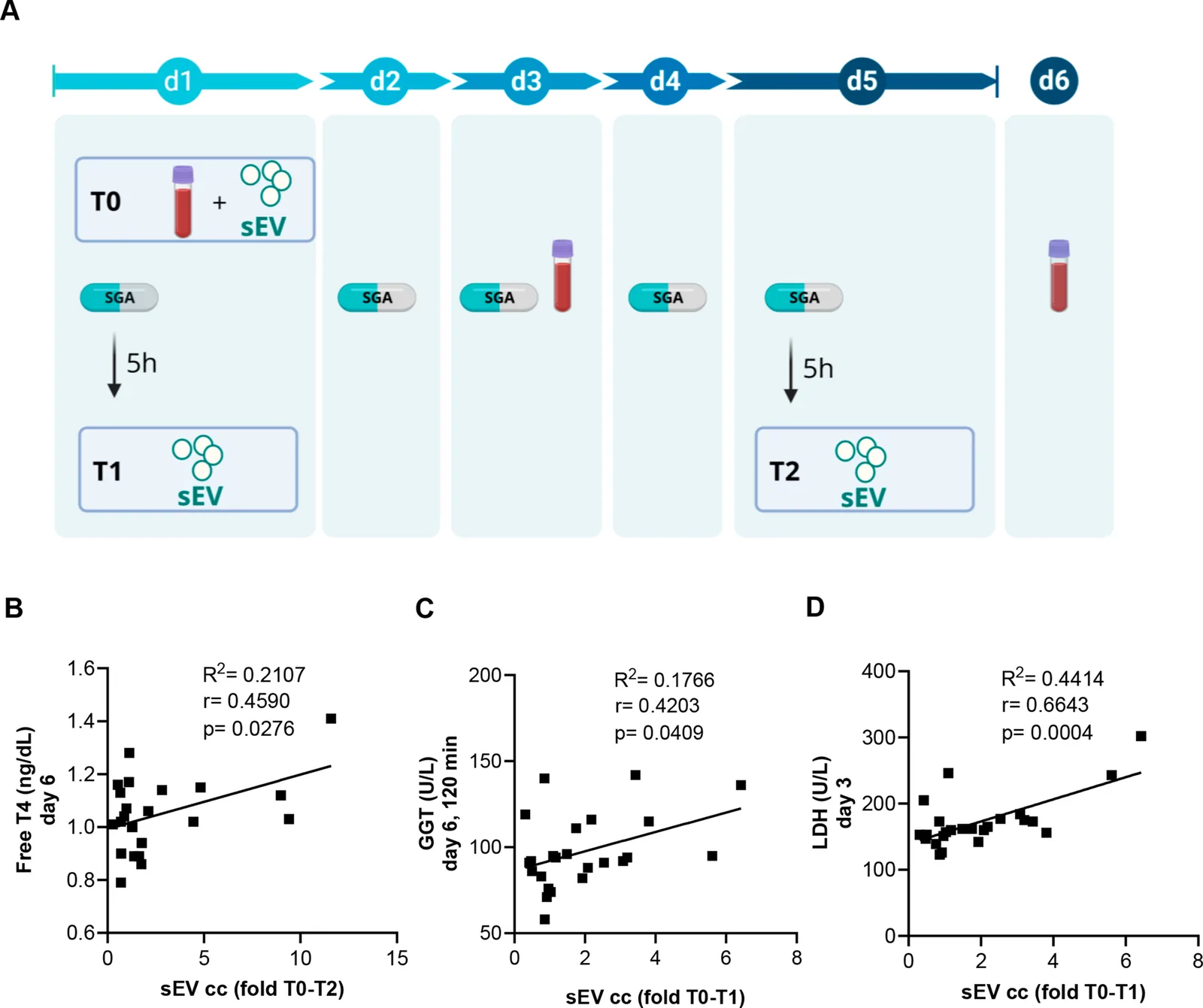
Correlations between changes in sEV concentration and metabolic or biochemical parameters in healthy volunteers treated with ARI. **A** Representative scheme of SGA dosing and blood collection. Image was performed in biorender.com. **B** Correlation between sEV concentration (fold change T0-T2) and free T_4_ (mg/dL) levels analysed at day 6 (*n* = 24). **C** Correlation between sEV concentration (fold change T0–T1) and GGT (U/L) analysed at day 6 (120 min) (*n* = 24). **D** Correlation between sEV concentration (fold change T0-T1) and LDH (U/l) determined on day 3 (*n* = 24). Each plot displays the regression line along with R², *r*, and *p* values indicating the strength and significance of the association. Pearson’s correlation test was used

**The amount of circulating sEV in healthy volunteers receiving ARI positively correlates with clinical parameters of metabolic alterations.** To determine whether the increase in circulating sEV following ARI administration was associated with biochemical markers of metabolic dysfunction, which were previously reported to be altered in first-episode psychosis and in patients treated with SGAs [28, 29], we examined the relationship between sEV abundance and relevant metabolic readouts. The scheme showing SGA dosing and blood collection is depicted in Fig. 2A.

As shown in Fig. 2B, in healthy volunteers, the rise in circulating sEV in the T0-T2 period (5 days after dosing) positively correlated with free T_4_ levels measured at day 6. Gamma-glutamyl transferase (GGT) activity also measured on day 6 was positively associated with the increase of sEV abundance at 5 h post-dosing (T0-T1) (Fig. 2C). Moreover, lactate dehydrogenase (LDH) activity, determined on day 3, correlated with sEV elevations at T0-T1 period (Fig. 2D). Notably, no statistically significant associations between the increase in sEV (both at T0-T1 and T0-T2) and insulin or C peptide (determined at day 6) were found. Likewise, no correlation was found between the increase in sEV abundance in the T0-T2 period with LDH activity analysed at day 3 (Figure S3). Regarding volunteers receiving OLA, no significant correlations with biochemical parameters were found (Figure S4).

**Fig. 3:**
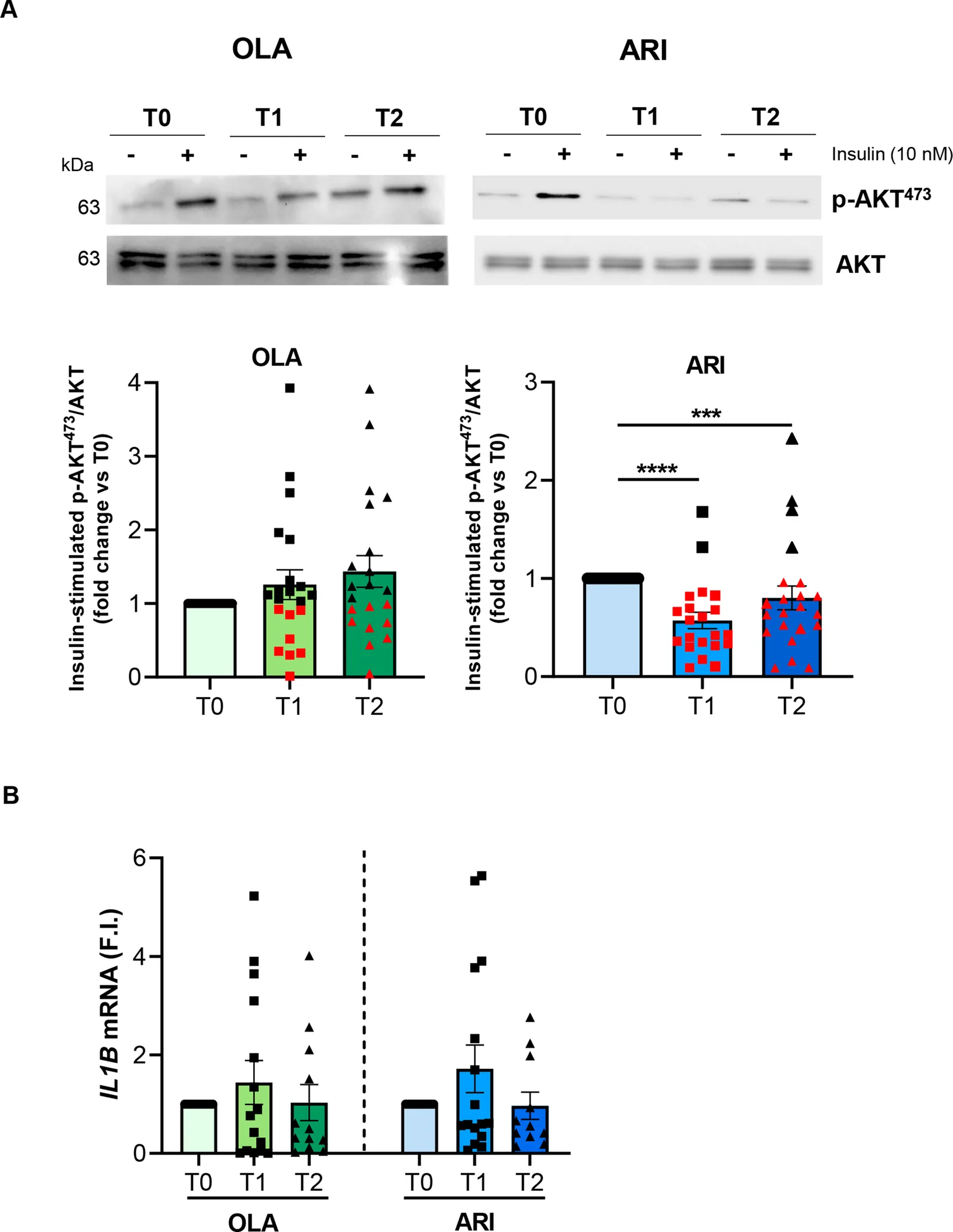
Insulin signalling and inflammatory response in healthy volunteers treated with OLA or ARI. **A** Representative Western blot of phosphorylated AKT at Ser473 (p-AKT^473^) and total AKT in Huh7 hepatocytes stimulated with insulin (10 nM) after 24 h exposure to sEV isolated from OLA and ARI treated volunteers at time points T0, T1, and T2. Quantification of the insulin-stimulated p-AKT^473^/AKT ratio is expressed as fold change relative to T0 (*n* = 21–23). The red dots indicate sEV-treated hepatocytes in which insulin-stimulated p-AKT^473^ induction was lower than the basal (T0) level, corresponding to a fold change < 1 and therefore classified as impaired insulin signalling. **B ***IL1B* mRNA expression levels in isolated PBMCs from OLA and ARI treated volunteers at T0, T1, and T2 (*n* = 11–16). Data are presented as mean ± SEM. In **A**: ***p < 0.001; ****p < 0.0001, One-way ANOVA, Kruskal-Wallis test

**Circulating sEV from healthy volunteers treated with ARI impaired insulin signalling in human Huh7 hepatocytes.** To evaluate the functional impact of circulating sEV on metabolically relevant cells, Huh7 human hepatocytes were incubated for 24 h with sEV isolated from volunteers at baseline (T0), T1 (5 h) or T2 (5 days) after SGA administration. Cells were subsequently stimulated with insulin (10 nM) for the final 10 min, and insulin responsiveness was assessed by measuring AKT phosphorylation at Ser473. As shown in Fig. 3A, hepatocytes exposed to sEV isolated prior to SGA administration (T0) exhibited increased AKT phosphorylation in response to insulin. Notably, sEV derived from ARI-treated volunteers markedly blunted this response as assessed by densitometric quantification of the Western blots, reducing insulin signalling in approximately 90.5% of the cases at T1 and 81.8% at T2, compared to the response at T0. As this inhibitory effect was evident at both early and later time points, these results suggest that the impairment of hepatocyte insulin signalling induced by sEV occurs rapidly following SGA exposure and that this dysfunction persists throughout the treatment duration. In contrast, sEV from OLA-treated volunteers impaired insulin signalling only in 38.1% of the cases at T1 and 42.8% at T2 compared to T0, consistent with the milder increase in circulating sEV concentration with this SGA.

To assess whether these effects were associated with systemic inflammation, *IL1B* mRNA levels were measured in PBMCs from the same volunteers across T0-T2. As shown in Fig. 3B, a trend towards an elevation was observed with both SGA at T1, although it was not statistically significant, pointing that the impairment of hepatocyte insulin signalling induced by circulating sEV is not accompanied by overt systemic inflammation. In line with this, no significant correlations were detected between circulating sEV and *IL1B* expression levels in PBMCs nor between *IL1B* expression in PBMCs and insulin-stimulated pAKT in sEV-treated hepatocytes (Figure S5).

**Fig. 4:**
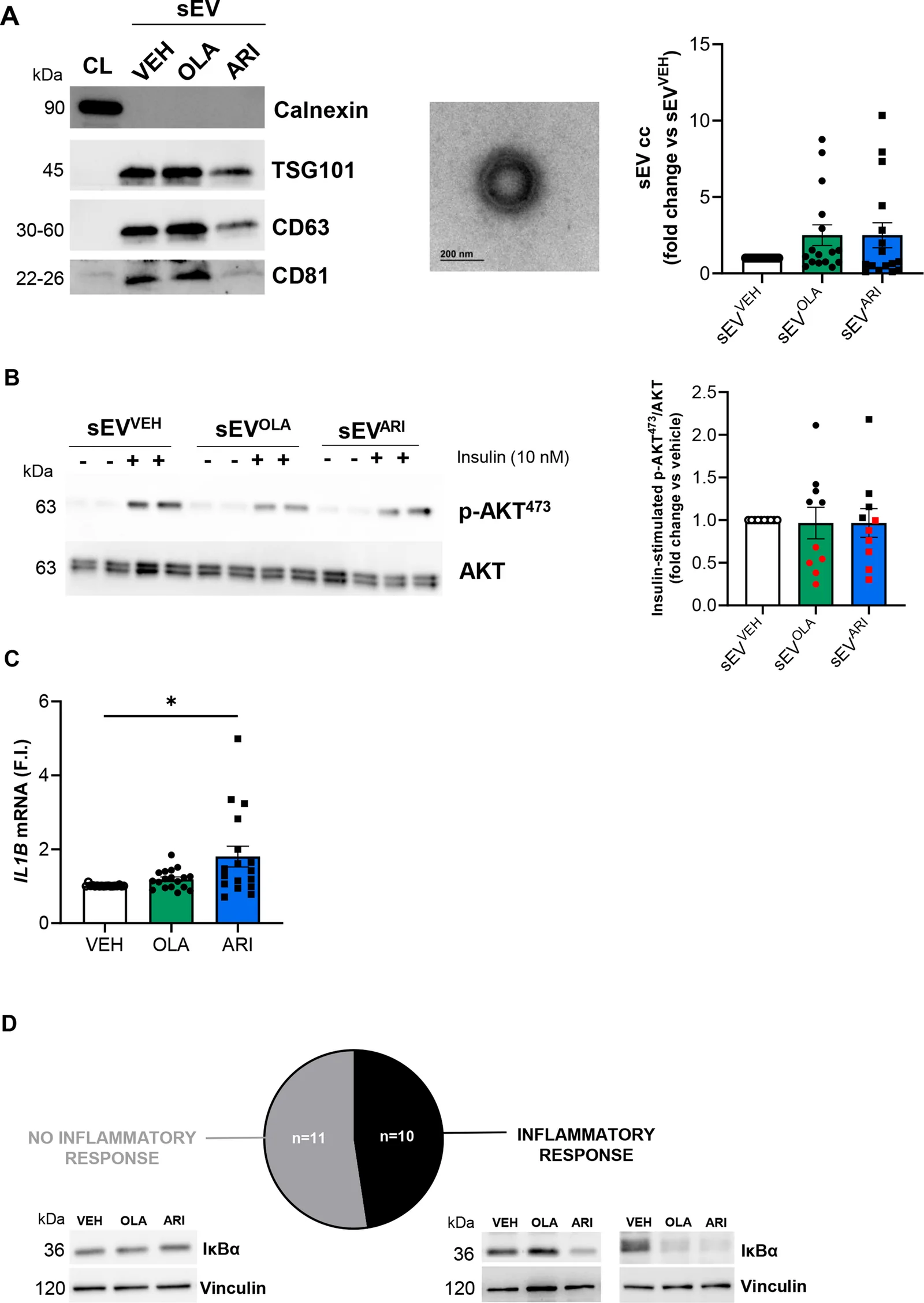
Functional effects of sEV secreted by human macrophages treated in vitro with OLA or ARI. **A** Representative Western blot analysis of sEV markers TSG101, CD63, and CD81 across treatment conditions. Calnexin was included as a negative control to exclude contamination of sEV preparations with cellular debris. *CL *cell lysate; *VEH *vehicle. A representative TEM image shows typical sEV morphology (scale bar: 200 nm). The bar graph shows sEV concentration measured by NTA and expressed as fold change relative to sEV^VEH^ (*n* = 16) **B** Representative Western blot of p-AKT^473^ and total AKT in response to insulin (10 nM) in Huh7 cells exposed to sEV^VEH^, sEV^OLA^, or sEV^ARI^. Quantification of the insulin-stimulated p-AKT^473^/AKT ratio is expressed as fold change relative to the basal condition (VEH) (*n* = 10). The red dots indicate the sEV-treated hepatocytes in which insulin-stimulated p-AKT^473^ induction was lower than the basal level (fold change < 1), corresponding to impaired insulin signalling. **C ***IL1B* mRNA expression levels under VEH, OLA, and ARI conditions (*n* = 17–18). **D** The pie chart summarizes the number of h-mac batches from healthy donors displaying no inflammatory response or an inflammatory response to SGAs. Representative IκBα Western blots depict the three response patterns observed in h-mac (*n* = 21): no response, response to ARI, and response to ARI and OLA. Data are presented as mean ± SEM. In **C**: *p < 0.05, One-way ANOVA, Kruskal-Wallis test

**sEV released by human macrophages exposed to OLA or ARI in vitro attenuate insulin signalling in human Huh7 hepatocytes.** To investigate whether h-mac contribute to the effects induced by SGAs on insulin signalling and inflammation by the release of sEV into circulation, cultured h-mac isolated from healthy donors were treated for 24 h with DMSO (VEH), OLA or ARI, and sEV were subsequently isolated from the culture medium. As shown in Fig. 4A, Western blot analysis demonstrated a robust expression of the canonical sEV markers TSG101, CD63, and CD81. In contrast, calnexin, an endoplasmic reticulum protein used as a negative control, was not detected in any preparation, indicating the absence of cellular contamination. TEM revealed round, membrane-bound vesicles with sizes consistent with sEV populations, confirming their structural integrity. Complementary NTA showed comparable size distribution profiles across VEH, OLA, and ARI conditions, with similar mean and modal diameters and no evidence of secondary peaks or large aggregates, supporting the homogeneity of the vesicle preparations (Figure S6). Quantitative analysis further indicated a non-significant trend to an increase in sEV concentration regardless of SGA exposure (Fig. 4A, right panel), suggesting that short-term exposure to OLA or ARI may not be sufficient to enhance vesicle release by h-mac in vitro.

Following 24 h incubation with sEV, insulin-stimulated AKT phosphorylation was preserved in Huh7 hepatocytes treated with sEV derived from VEH-treated h-mac. In contrast, AKT phosphorylation was reduced by 50% or 60% when Huh7 hepatocytes were exposed to sEV released by OLA- or ARI-treated h-mac, respectively, as assessed by densitometric quantification of the Western blots (Fig. 4B). Further analysis of *IL1B* mRNA expression in h-mac revealed a significant increase following ARI treatment (Fig. 4C). We next analysed IκBα protein levels in h-mac treated with SGAs, and representative Western blots are shown. Across all donors (*n* = 21), 11 h-mac did not respond to either OLA or ARI in inducing an inflammatory response defined by the decrease in IκBα protein levels relative to vehicle (VEH, DMSO) (representative Western blot shown in Fig. 4D, left). In contrast, 10 donors showed an inflammatory response to ARI evidenced by IκBα reduction, whereas only 4 donors responded to OLA with similar decrease (representative Western blots shown in Fig. 4D, right). Figure S7 shows that neither sEV concentration nor pAKT Ser473 levels correlated with *IL1B* expression in OLA- or ARI-treated h-mac, as no significant associations were detected between these variables. Collectively, the data support the notion that h-mac are a relevant source of sEV in response to SGA exposure, and that the capacity of these vesicles to impair insulin signalling depends on both the specific SGA and donor-intrinsic factors.

**Fig. 5:**
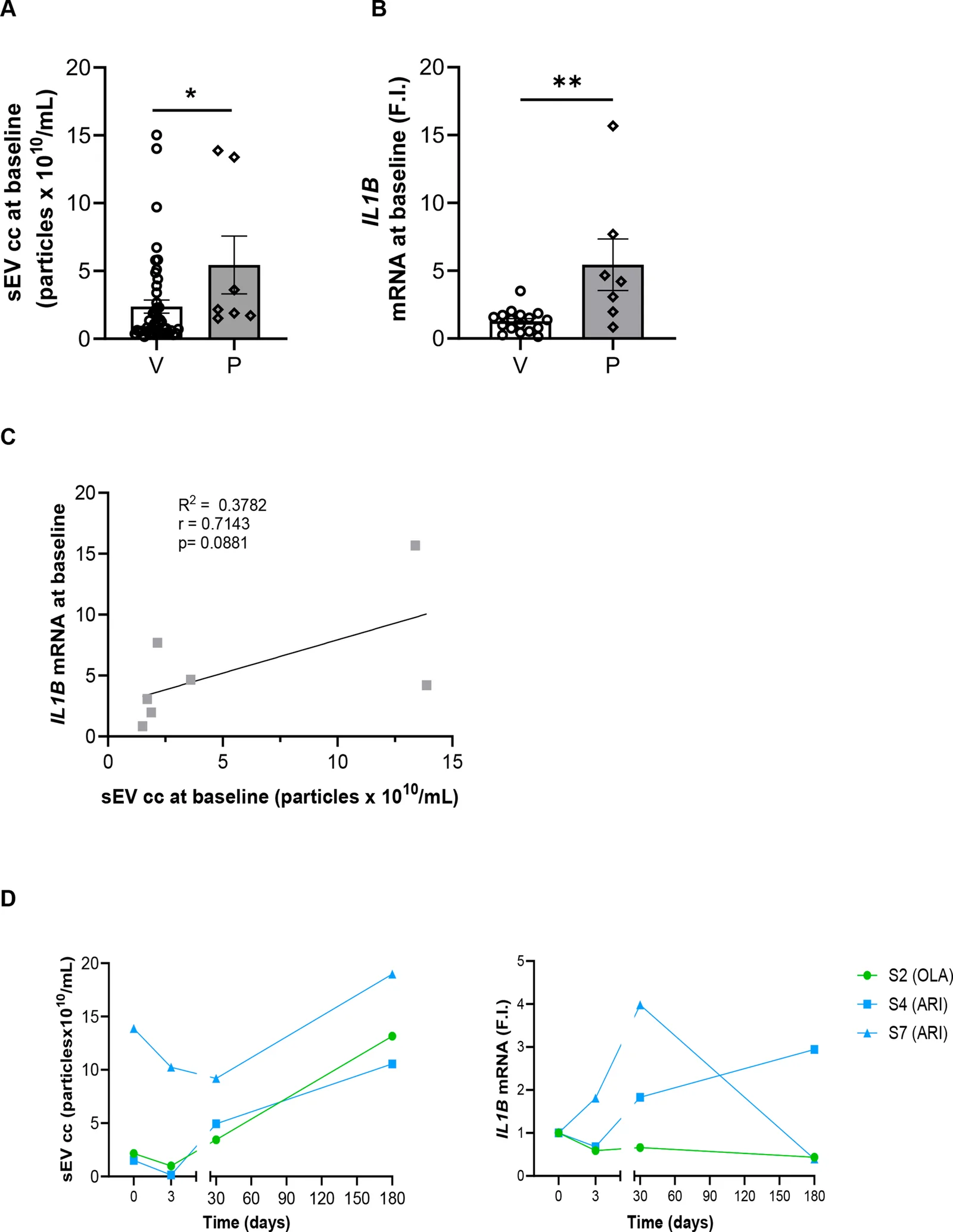
Basal sEV concentration and baseline *IL1B* mRNA expression in healthy volunteers (V) and patients with first-episode psychosis (P). **A** Quantification of sEV concentration (particles × 10^10^/mL) at baseline, in healthy volunteers (V, *n* = 48) and patients with first-episode psychosis (P, *n* = 7). **B** Baseline *IL1B* mRNA expression levels were measured in PBMCs from the same groups (*n* = 17 volunteers, *n* = 7 patients). **C** Correlation analysis between sEV concentration and *IL1B* mRNA expression at baseline across all participants. **D** Longitudinal trajectories of circulating sEV concentration (particles × 10^10^/mL) and *IL1B* mRNA expression monitored in three representative patients (S2: OLA, S​4: ARI, S7: ARI) across the 6-month follow-up period (T0–T180 days). Data are presented as mean ± SEM. In **A** and **B**: *p < 0.05; **p < 0.01, Mann–Whitney U test. In **C**: association evaluated using Spearman’s correlation

**Circulating sEV levels were increased in patients with first-episode psychosis**,** and this increase was enhanced upon SGA treatment.** Next, circulating sEV were examined in seven patients experiencing a first-episode psychosis subsequently treated with OLA or ARI and followed for six months. Body weight and BMI at baseline are shown in Table S2. We first compared sEV concentration between healthy volunteers participating in this study and the individuals experiencing a first episode of psychosis at baseline (before SGA treatment). As shown in Fig. 5A, patients diagnosed with a first episode of psychosis (*n* = 7) exhibited significantly higher levels of circulating sEV than healthy volunteers at baseline. Notably, this elevation in sEV abundance was accompanied by increased *IL1B *mRNA expression in PBMCs (Fig. 5B), suggesting an association between enhanced sEV release and low-grade inflammatory activation in these patients. Consistent with this interpretation, sEV concentration positively correlated with *IL1B *expression (Fig. 5C), supporting a potential link between inflammatory status and increased sEV release in early psychosis.

Finally, as an exploratory approach, we analysed sEV concentrations in the circulation of three of the patients who could be monitored for up to six months of SGA treatment (one patient with OLA and two patients with ARI). As shown in Fig. 5D, the three SGA-treated patients exhibited elevated circulating sEV concentrations after six months of treatment compared with their respective baseline levels. Interestingly, only the two patients treated with ARI showed elevated *IL1B* expression in PBMCs: one presented a peak at month 1 that declined thereafter, while the other maintained high *IL1B* expression levels at month 6. These findings might suggest that both a first psychotic episode and ARI treatment might increase the risk of systemic inflammation linked to elevated circulating sEV levels.

## Discussion

Multiple meta-analyses have shown that individuals with schizophrenia exhibit impaired glucose homeostasis, elevated fasting insulin, and increased HOMA-IR even before antipsychotic exposure, indicating an intrinsic predisposition to metabolic dysfunction [30–32]. Additionally, SGAs further exacerbate this vulnerability, with OLA exerting the greatest metabolic burden and ARI producing milder, yet still clinically relevant, effects [33, 34]. The convergence of disease-related and drug-related metabolic stressors generates a cumulative risk for insulin resistance and type 2 diabetes. Although previously conducted mechanistic preclinical studies by us [35–38] and others (reviewed in [28, 39]) have provided insight into the metabolic disturbances induced by the treatment with ARI and OLA, the potential mediators that transmit signals able to trigger inflammation and alter insulin sensitivity in metabolically relevant cells remain poorly defined.

Growing evidence from human studies implicates circulating sEV in metabolic diseases. For example, sEV derived from adipose tissue, liver, skeletal muscle, and immune cells have been shown to contribute to the development of insulin resistance, metabolic syndrome, and type 2 diabetes [40, 41]. Notably, the sEV cargo, particularly miRNAs and inflammatory mediators, are able to predict the severity of insulin resistance and future metabolic outcomes [42–44]. In this context, herein we show that ARI significantly increased the amount of sEV in circulation in healthy volunteers, and that this increase was positively associated with markers of endocrine disorders (free T_4_) and liver disease (GGT and LDH). Importantly, the circulating sEV isolated from healthy volunteers following exposure to ARI were able to impair insulin-stimulated AKT phosphorylation in Huh7 human hepatocytes, indicating that these vesicles are not merely byproducts of the treatment, but rather act as active mediators capable of transmitting insulin-desensitizing signals. However, the effect of OLA on increasing the pool of circulating sEV, and its subsequent impact in hepatocyte insulin signalling, was less pronounced. These differences point to SGA-dependent responses in modulating sEV release and their capacity to alter hepatocyte insulin signalling, which may be clinically relevant when prescribing SGAs, particularly for individuals with pre-existing insulin resistance. It is noteworthy to highlight that Huh7 is a hepatoma-derived line and the extrapolation of the results to primary hepatocytes and to in vivo hepatic insulin sensitivity is a limitation of our study.

Although OLA is classically associated with more severe metabolic side-effects than ARI, this cannot be generalized to all clinical contexts. For instance, a recent longitudinal study in 422 patients treated with ARI has reported metabolic adverse effects such as weight gain and elevation in blood lipids in a dose- and treatment duration-dependent manner [45]. Consistent with this, we have recently reported that both SGAs induced weight gain and beta cell dysfunction in female mice, leading to glucose intolerance. However, ARI had a more potent effect in interfering with calcium signalling in beta cells, which was likely a result of its ability to modulate the serotonergic system in pancreatic islets [35] suggesting that other molecular mechanisms might explain the metabolic side effects observed during the treatment with this SGA.

Additional in vitro experiments conducted herein further implicate macrophages as a pathologically relevant source of sEV, as sEV released by cultured h-mac upon SGA treatment also impaired hepatocyte insulin signalling and, in this case, minor differences were found between both SGAs. Currently, we cannot assess the specific sEV cargo responsible for this effect, but we can speculate that, among the sEV cargo, miRNAs are potential effectors since they modulate the expression levels of critical nodes of the insulin signalling cascade. In this regard, it has recently been shown that exosomes released by bone marrow-derived macrophages from obese mice markedly suppressed AKT/GSK pathway activity and impaired glycogen synthesis in hepatocytes via miR-222-5p [46]. Additionally, reactive lipid species such as saturated free fatty acids transported in sEV can also impair insulin signalling in hepatocytes as previously reported by our group [47]. Therefore, future studies characterizing the molecular cargo of sEV from ARI- and OLA-treated healthy volunteers will be essential to identify the mediators driving these metabolic effects.

In psychiatry, emerging evidence suggests altered sEV profiles in schizophrenia, including immune-related and metabolic signatures [48], although this area remains unexplored. This immune-metabolic crosstalk is consistent with broader evidence describing chronic low-grade inflammation and altered immune signalling in schizophrenia. In fact, meta-analyses have reported elevations in IL-1β, IL-6, TNF-α, and other pro-inflammatory cytokines across disease stages [49, 50]. Importantly, similar inflammatory signatures have been associated with metabolic dysfunction and insulin resistance in SGAs-treated individuals [51, 52]. In our study, although results revealed a trend, without statistical significance, towards an increase in *IL1B* mRNA levels in h-mac from healthy volunteers receiving OLA or ARI, data obtained in cultured h-mac treated with SGAs revealed an increase in cells exposed to ARI. These discrepancies are likely due to differences in SGA concentrations, binding profiles and pharmacokinetics across experimental conditions, but they also raise the possibility that long-term SGA treatment might exacerbate systemic inflammation. This hypothesis is supported by the elevations in *IL1B* found in h-mac from two individuals with a first-episode psychosis treated with ARI for up to six months that also displayed elevated concentrations of sEV in circulation. Although our patient cohort is very limited due to challenges in longitudinal monitoring, the exploratory results presented herein, if confirmed in larger studies, may help to identify individuals at risk of SGA-associated metabolic disturbances. Another result that supports SGA-mediated systemic inflammation is the decrease in IκBα protein levels in h-mac from healthy donors treated with SGAs in vitro, an effect particularly evident in the case of ARI. The convergence of results across healthy volunteers, donor h-mac, and patients strengthens the fact that SGAs are potential triggers of inflammatory signatures likely by favoring sEV release by h-mac that might modulate distant responses in peripheral tissues. In fact, immune cells such as macrophages release sEV in multiple pathological contexts, including metabolic diseases as reviewed [53].

## Conclusions

The findings of the present study position sEV as mechanistic contributors to the early metabolic alterations induced by SGAs, providing a potential functional link between immune cell activity and vesicle-mediated signalling, and hepatocyte insulin resistance. The observation that ARI and OLA differentially modulate sEV release, as well as their differential dysregulation of insulin signalling in Huh7 hepatocytes, highlights the importance of drug-specific effects on vesicle biology, with implications for understanding the variable metabolic side-effect profiles of these SGAs. Collectively, our results advance the concept that sEV are not passive biomarkers but represent active effectors of SGA-induced metabolic dysregulation, offering novel avenues for mechanistic studies and potential therapeutic targeting.

## Supplementary Information

Supplementary Material 1.

Supplementary Material 2.

## Data Availability

Experimental data generated in this study are available from the corresponding authors upon reasonable request.
