# Supplementary material for: Exposure to second-generation antipsychotics increases circulating small extracellular vesicles that promote insulin resistance in hepatocytes
Source: J Physiol Biochem. 2026 Nov 3;82(1):111. doi: 10.1007/s13105-026-01250-7 (PMC13731531; doi:10.1007/s13105-026-01250-7)
Supplement: Supplementary file 1 [file 13105_2026_1250_MOESM2_ESM.pdf]

Figure 1A

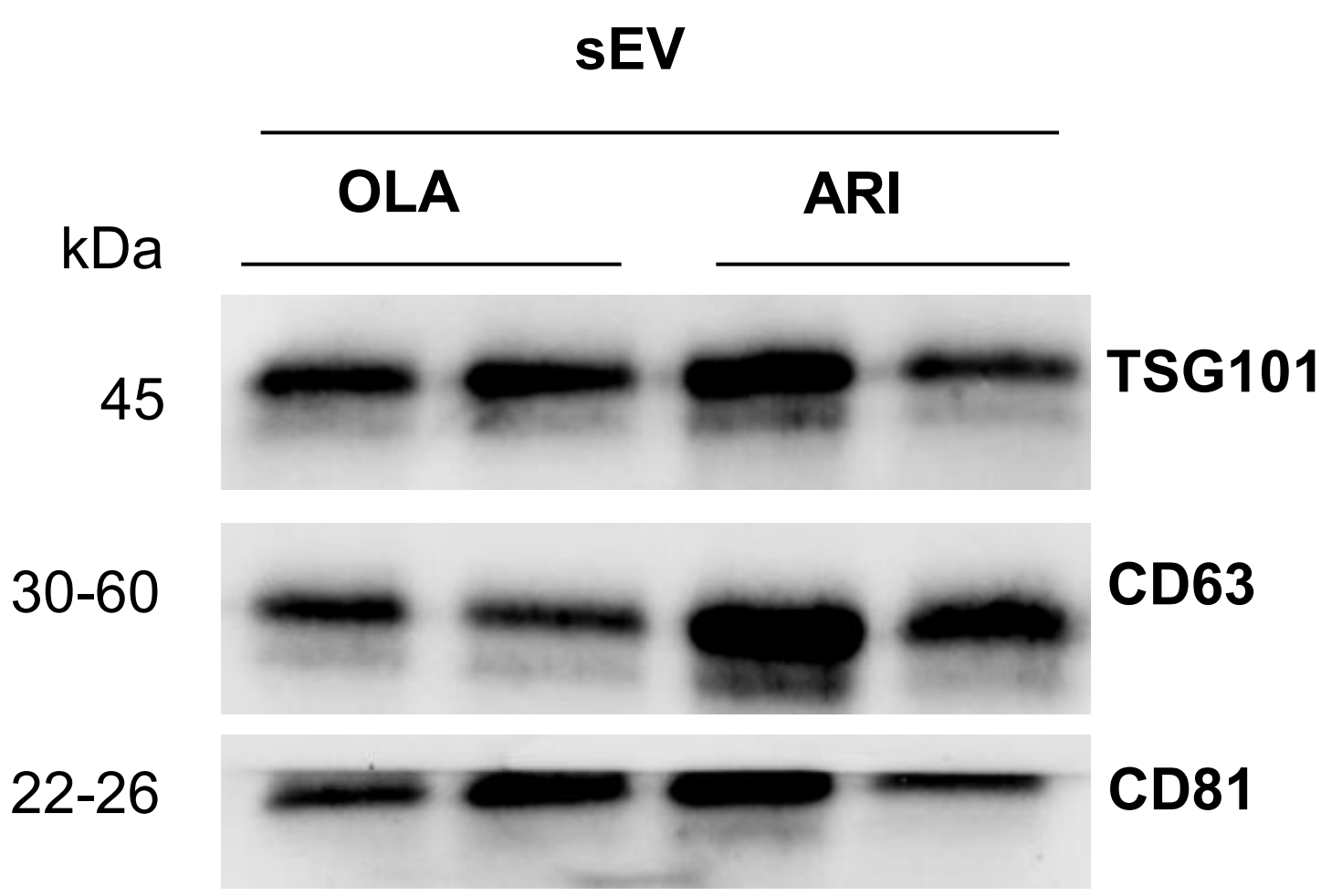

sEV
OLA
ARI
kDa
45
30-60
22-26
TSG101
CD63
CD81

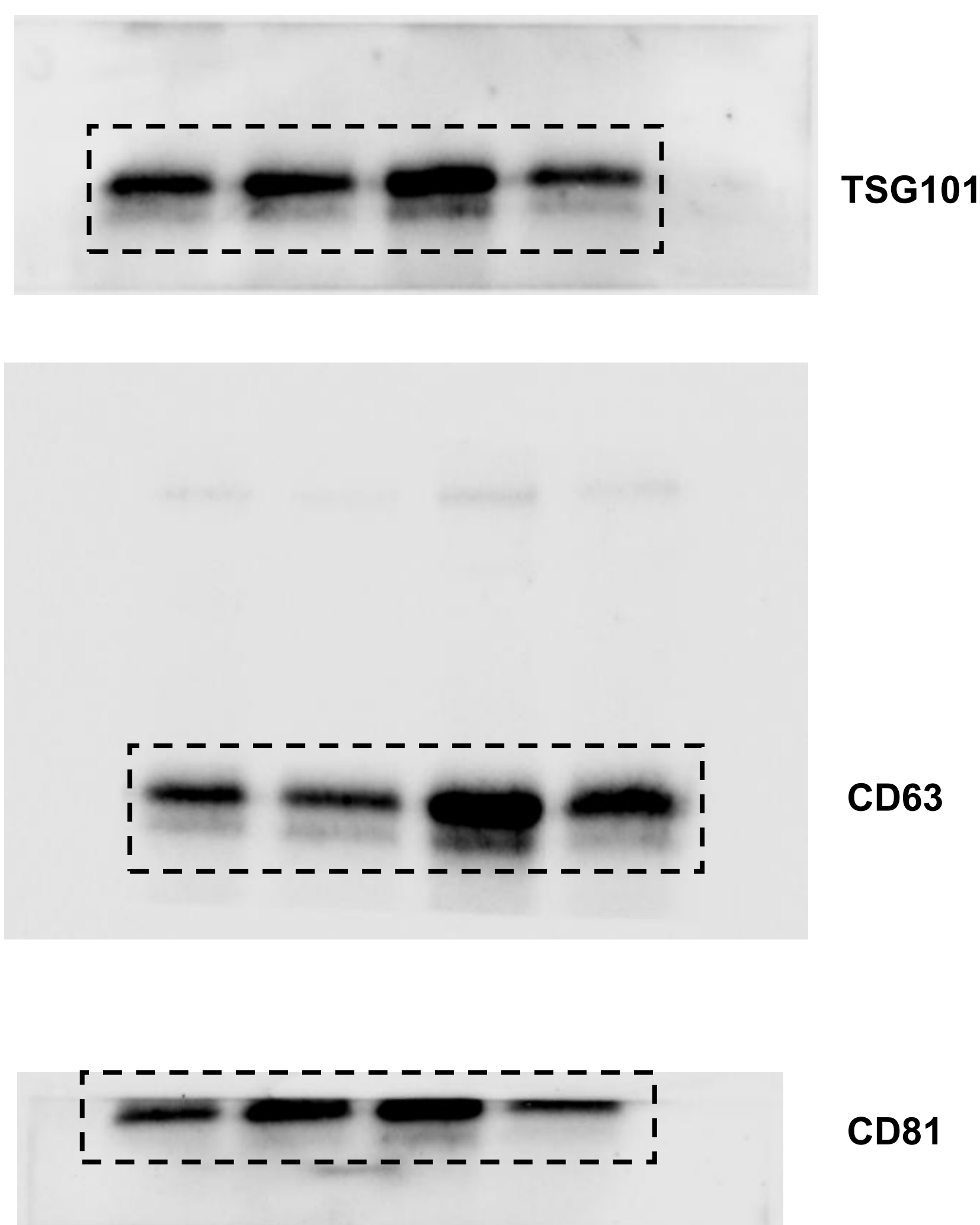

TSG101
CD63
CD81

Figure 3A

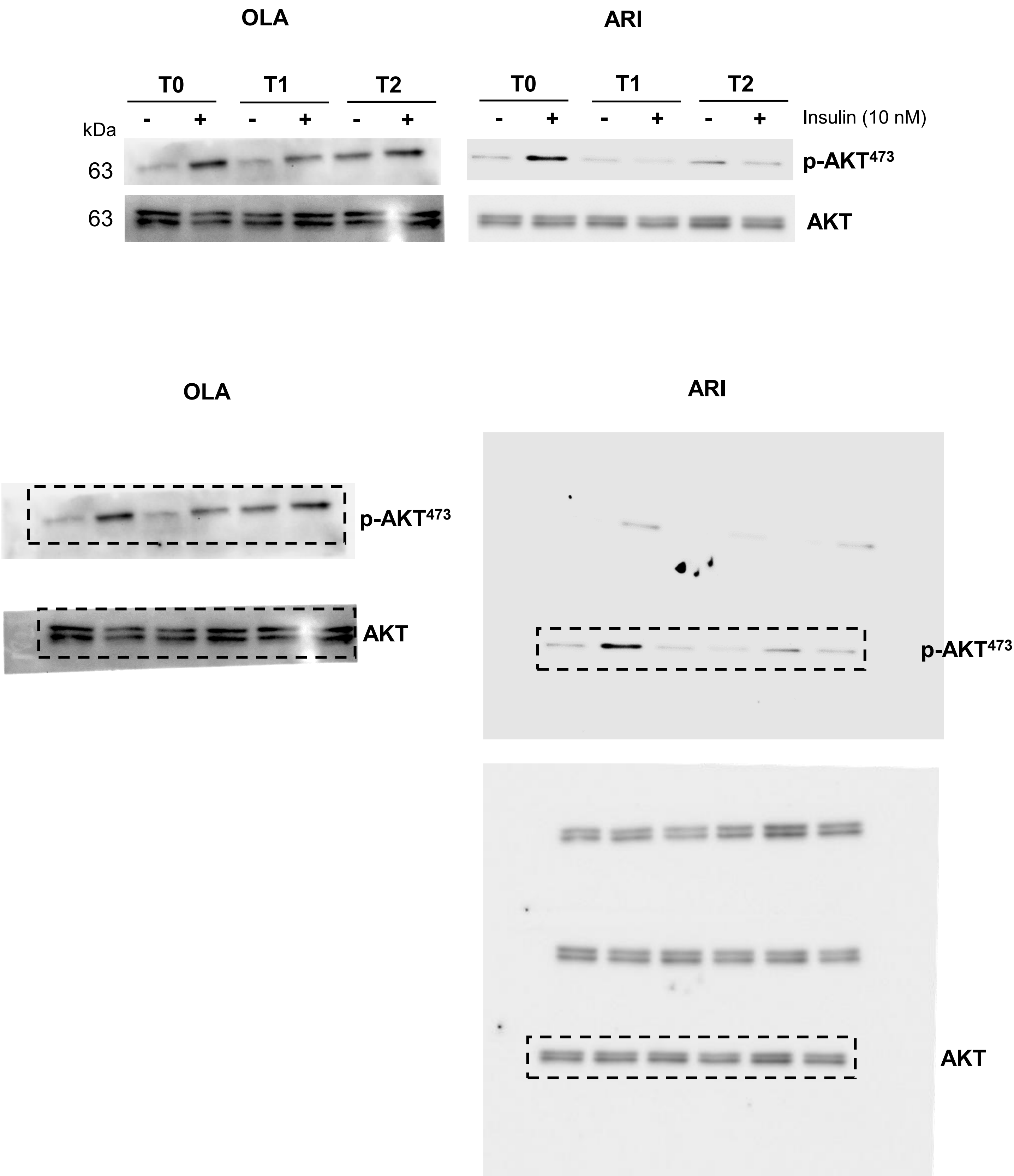
OLA
ARI
T0
T1
T2
T0
T1
T2
kDa
- + - + - +
- + - + - +
Insulin (10 nM)
63
63
p-AKT473
AKT
OLA
ARI
p-AKT473
AKT
p-AKT473
AKT

Figure 4A

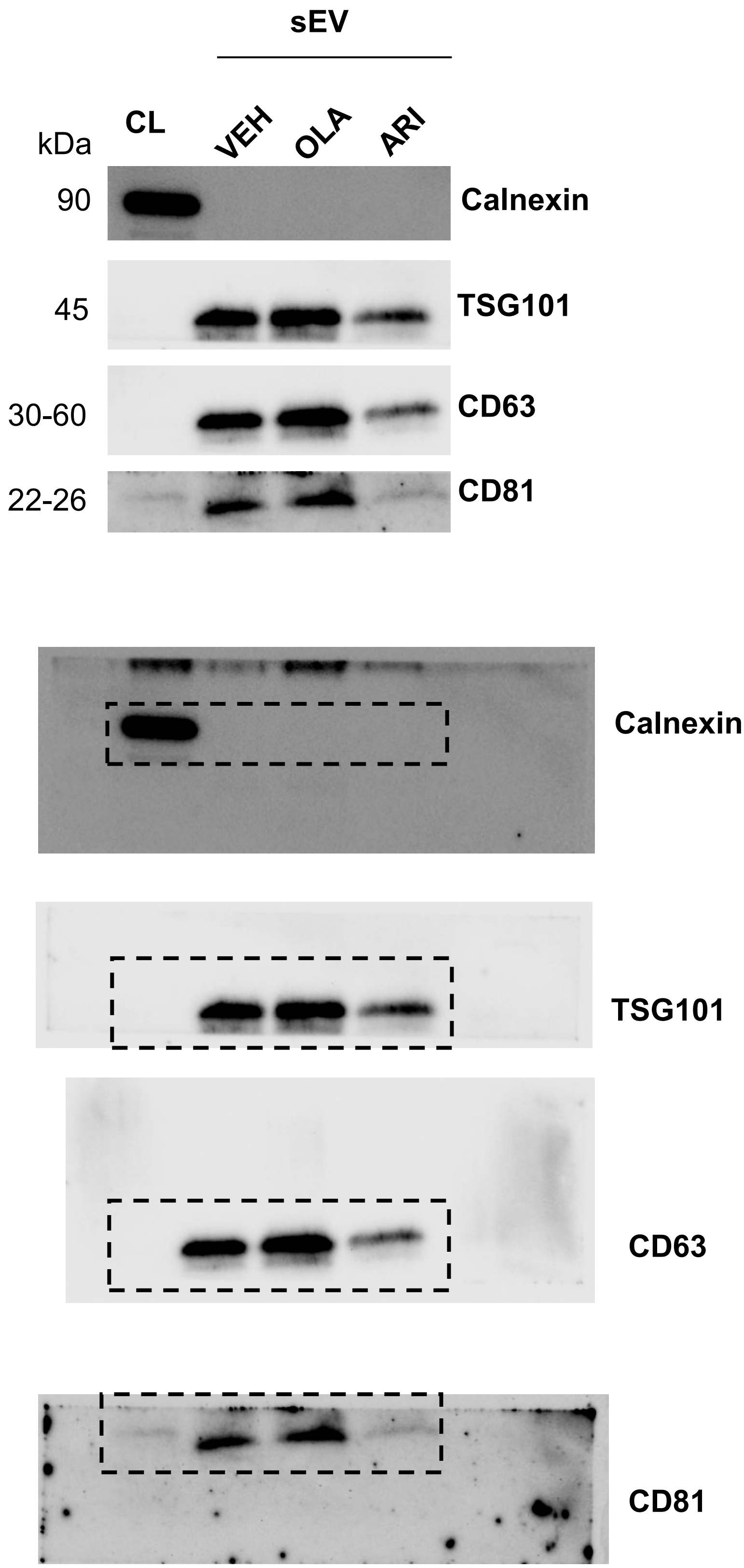

sEV
CL
VEH
OLA
ARI
kDa
90
Calnexin
45
TSG101
30-60
CD63
22-26
CD81
Calnexin
TSG101
CD63
CD81

Figure 4B

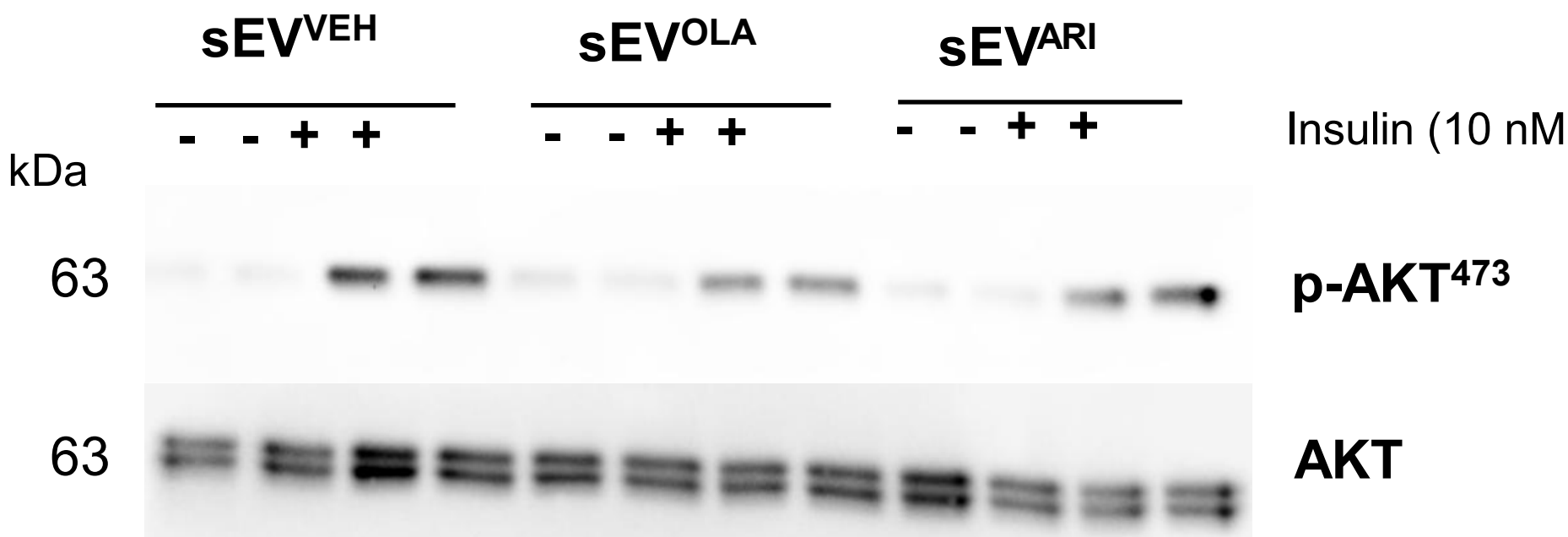
sEV^VEH
sEV^OLA
sEV^ARI
- - + + - - + + - - + +
Insulin (10 nM)
kDa
63
p-AKT^473
63
AKT

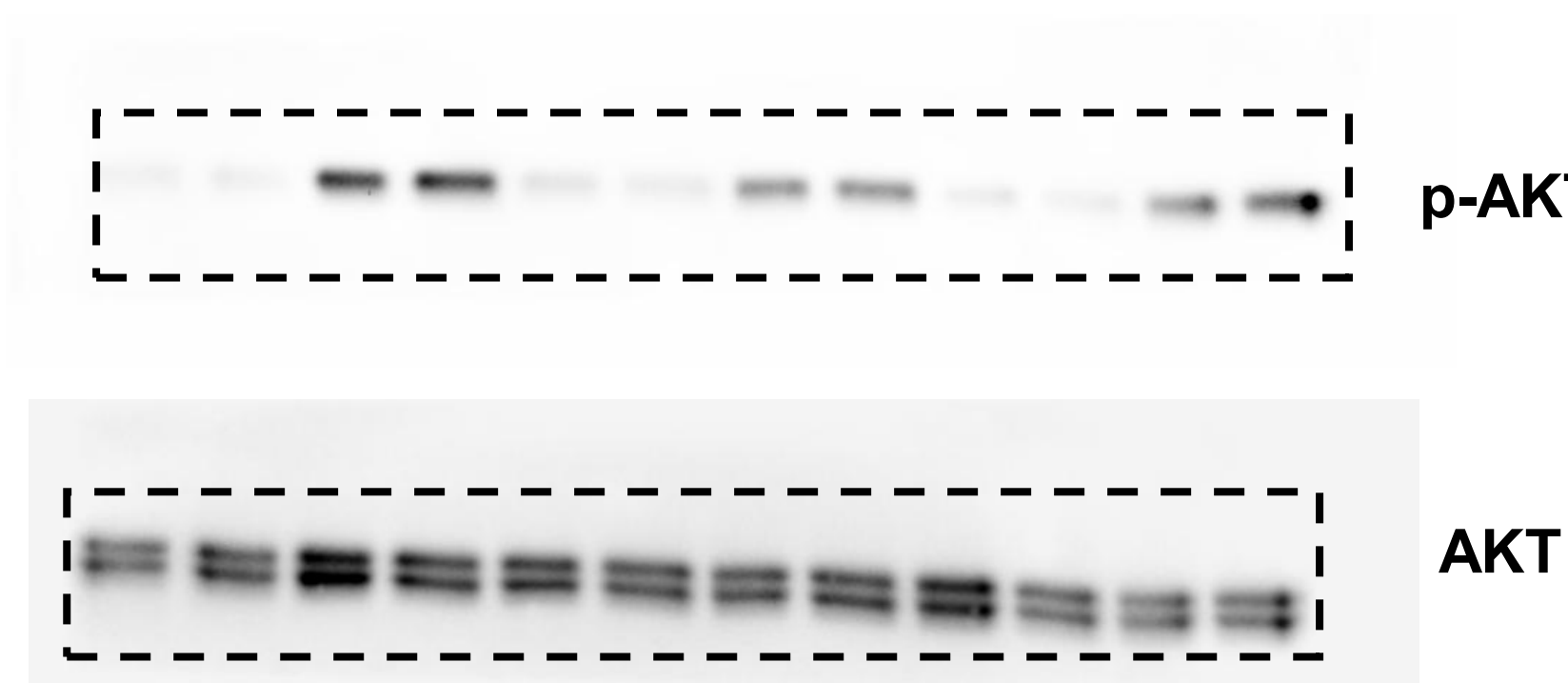
p-AKT^473
AKT

Figure 4D

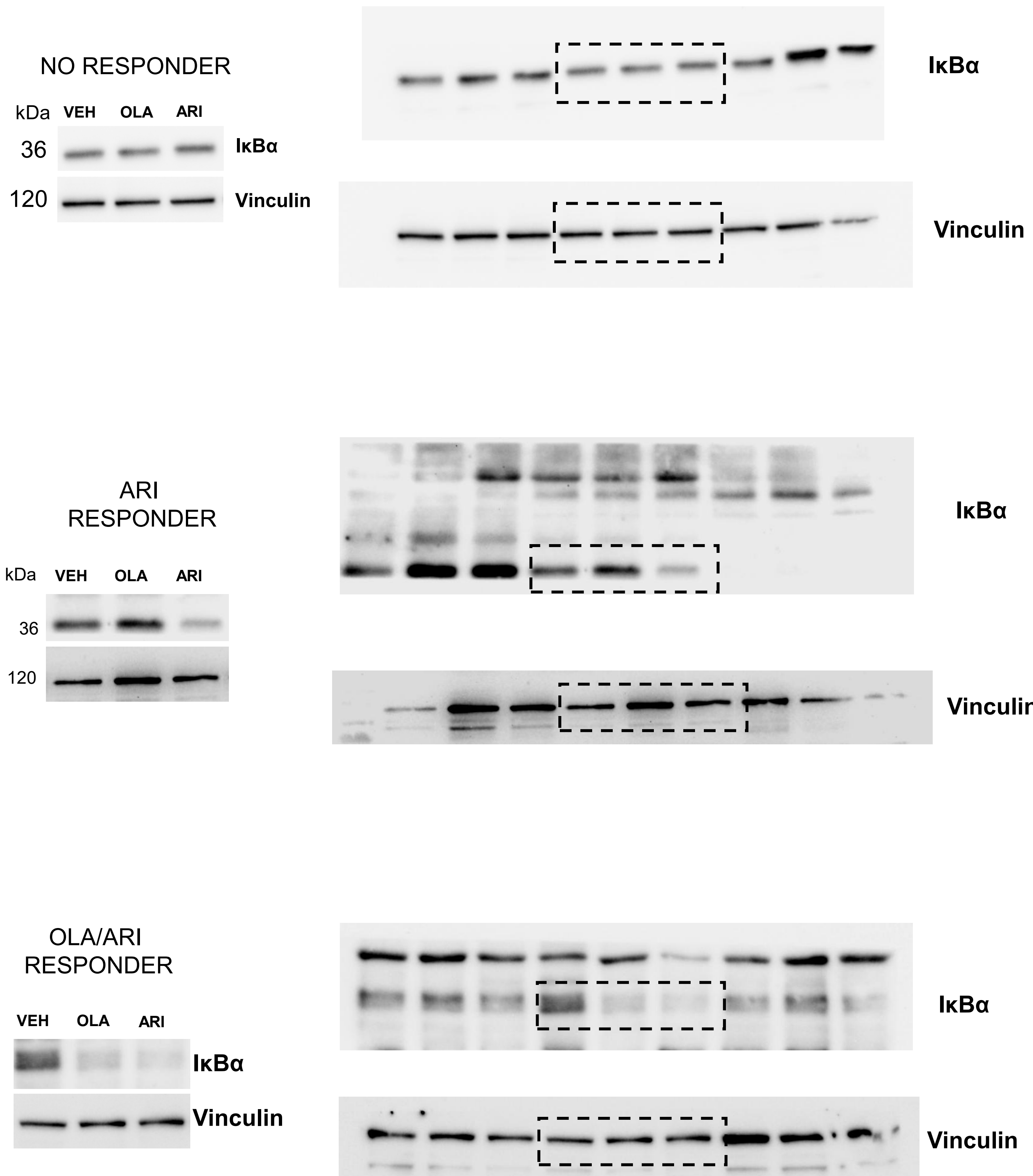
NO RESPONDER
kDa
VEH
OLA
ARI
36
IκBα
120
Vinculin
IκBα
Vinculin
ARI RESPONDER
kDa
VEH
OLA
ARI
36
120
IκBα
Vinculi
OLA/ARI RESPONDER
VEH
OLA
ARI
IκBα
Vinculin
IκBα
Vinculin
