# Supplementary material for: Exposure to second-generation antipsychotics increases circulating small extracellular vesicles that promote insulin resistance in hepatocytes
Source: J Physiol Biochem. 2026 Nov 3;82(1):111. doi: 10.1007/s13105-026-01250-7 (PMC13731531; doi:10.1007/s13105-026-01250-7)
Supplement: Supplementary file 2 [file 13105_2026_1250_MOESM1_ESM.docx]

**Exposure to Second-Generation Antipsychotics increases Circulating Small Extracellular Vesicles that Promote Insulin Resistance in Hepatocytes**

Vitor Ferreira, Rosa Alén, Dora Koller, Adrián Povo-Retana, Diana Grajales, Yolanda Sanchez-Carro, Lisardo Boscá, Manuel Izquierdo, Pilar López-García, Paz Garcia-Portilla, Francisco Abad-Santos, Irma Garcia-Martinez and Ángela M. Valverde.

**Includes:**

[**Supplementary Figures**	2](#_Toc226472379)

[**Supplementary Tables**	11](#_Toc226472380)

# **Supplementary Figures**

**Figure S1. Sex‑based comparison of circulating sEV concentration.** Circulating sEV levels were quantified in healthy volunteers treated with OLA (n = 22; 11 males and 11 females) or ARI (n = 24; 12 males and 12 females). For each treatment group, individual data points corresponding to male and female participants are displayed separately, together with the mean ± SEM.

**Figure S2. Size distribution profiles of circulating sEV from healthy volunteers treated with OLA or ARI.** Representative Nanoparticle Tracking Analysis (NTA) size‑distribution curves of plasma sEV collected at T0, T1, and T2 from healthy volunteers undergoing olanzapine (OLA; upper panels) or aripiprazole (ARI; lower panels) treatment. For each time point and treatment, the mean particle size, modal size, and standard deviation (SD) obtained from the corresponding NTA measurements are shown. Shaded areas indicate variability across samples. These panels illustrate typical examples of the size‑distribution curves obtained for each condition.

**Figure S3. Correlation analyses between changes in plasma sEV concentration and metabolic or biochemical parameters in healthy volunteers treated with ARI. (A)** Correlation between sEV concentration (fold change T0-T1 (n=24) or T0-T2 (n=23)) and insulin levels (µU/mL) on day 6. **(B)** Correlation between sEV concentration (fold change T0-T1 (n=24) or T0-T2 (n=24)) and C‑peptide levels (ng/mL) on day 6. **(C)** Correlation between sEV concentration (fold change T0-T2) and LDH levels (U/L) on day 3 (n=23). Each scatter plot includes a regression line and statistical parameters (R², *r*, and *p* value) indicating the strength and significance of the association. Correlations were evaluated using Pearson’s correlation when both normality and linearity criteria were met; otherwise, Spearman’s rank correlation was applied.

**Figure S4. Correlation analyses between changes in plasma sEV concentration and metabolic or biochemical parameters in healthy volunteers treated with OLA. (A)** Correlation between sEV concentration (fold change T0-T1 (n=21) or T0-T2 (n=23)) and insulin levels (µU/mL) on day 6. **(B)** Correlation between sEV concentration (fold change T0-T1 (n=22) or T0-T2 (n=23)) and C‑peptide levels (ng/mL) on day 6. **(C)** Correlation between sEV concentration (fold change T0-T2) and free T4 levels (ng/dL) on day 6 (n=22). **(D)** Correlation between sEV concentration (fold change T0-T1) and GGT levels (U/L) at 120 minutes on day 6 (n=23). **(E)** Correlation between sEV concentration (fold change T0-T1 (n=23) or T0-T2 (n=23)) and LDH levels (U/L) on day 3. Each scatter plot includes a regression line and statistical parameters (R², *r*, and *p* value) indicating the strength and significance of the association. Correlations were evaluated using Pearson’s correlation when both normality and linearity criteria were met; otherwise, Spearman’s rank correlation was applied.

**Figure S5. Correlation analyses among circulating sEV, insulin signalling, and inflammatory markers. (A)** Scatter plots depicting relationships between sEV concentration and *IL1B* mRNA expression in PBMCs across treatment groups and time points. For OLA: T0–T1 (n = 17) and T0–T2 (n = 12). For ARI: T0–T1 (n = 16) and T0–T2 (n = 12). **(B)** Scatter plots illustrating associations between *IL1B* Mrna EXPRESSION and insulin‑resistance-related signalling (pAKT^473^/AKT) in Huh7 cells for both treatments at T0-T1 and T0-T2. For OLA: T0–T1 (n = 14) and T0–T2 (n = 11). For ARI: T0–T1 (n = 14) and T0–T2 (n = 12). Each plot displays individual subject values together with linear regression lines and corresponding correlation statistics (r, R², p). All correlations were evaluated using Spearman’s rank correlation, as none of the datasets met normality or linearity criteria.

**Figure S6. Size distribution profiles of sEV released by human macrophages (h-mac) treated *in vitro* with VEH, OLA, or ARI.** Representative Nanoparticle Tracking Analysis (NTA) size‑distribution curves of sEV isolated from h-mac culture supernatants following *in vitro* treatment with vehicle (VEH), olanzapine (OLA), or aripiprazole (ARI). For each condition, the mean particle size, modal size, and standard deviation (SD) are reported. Shaded areas represent variability across samples. These panels illustrate typical examples of the size‑distribution curves obtained for each condition.

**Figure S7. Correlation analyses of sEV concentration, insulin signalling, and inflammatory markers relative to sEV^VEH^. (A)** Scatter plots depicting relationships between sEV concentration and *IL1B* mRNA expression in h-mac, expressed as fold change relative to sEV^VEH^, for both OLA (n=14) and ARI (n=11) treatments. **(B)** Scatter plots illustrating associations between *IL1B* mRNA expression in h-mac and pAKT^473^/AKT signalling in Huh7 cells, expressed as fold change relative to sEV^VEH^, under OLA (n=12) and ARI (n=11) conditions. Each plot displays individual subject values with linear regression lines and corresponding correlation statistics (r, R², p). Correlations were evaluated using Pearson’s correlation when both normality and linearity criteria were met; otherwise, Spearman’s rank correlation was applied.

# **Supplementary Tables**

**Table S1. Antibodies used in Western blot.**

| **Antibody** | **Supplier** | **Cat. no** | **Host specie** | **Dilution** |
| --- | --- | --- | --- | --- |
| Anti-AKT | Cell Signaling Technology | 4691 | Rabbit | 1:10000 |
| Anti-CALNEXIN | StressMarq Biosciences | SPC-108B | Rabbit | 1:1000 |
| Anti-CD63 | Santa Cruz | sc-5275 | Mouse | 1:1000 |
| Anti-CD81 | Santa Cruz | sc-166029 | Mouse | 1:1000 |
| Anti-IκBα | Santa Cruz | sc-371 | Rabbit | 1:1000 |
| Anti-Mouse | Santa Cruz | sc-516102 | Goat | 1:10000 |
| Anti-phospho-AKT | Cell Signaling Technology | 4058 | Rabbit | 1:1000 |
| Anti-Rabbit | Bethyl Laboratories | A120-108P | Goat | 1:20000 |
| Anti-TSG101 | Abcam | ab30871 | Rabbit | 1:1000 |
| Anti-VINCULIN | Santa Cruz | sc-73614 | Mouse | 1:20000 |

**Table S2. Baseline weight and BMI of individuals with first-episode psychosis.**

| **Subject ID** | **BMI (kg/m^2^)** | **Weight (Kg)** |
| --- | --- | --- |
| 1 | 27.70 | 73.6 |
| 2 | 20.34 | 58.1 |
| 3 | 26.35 | 60.1 |
| 4 | 33.31 | 71.5 |
| 5 | 22.42 | 67.1 |
| 6 | 34.95 | 109.5 |
| 7 | 18.35 | 54.4 |
